# Reduced cortical thickness in individuals with congenital adrenal hyperplasia (CAH)

**DOI:** 10.1038/s41598-026-45407-2

**Published:** 2026-03-25

**Authors:** Eileen Luders, Debra Spencer, Ieuan A. Hughes, Ajay Thankamony, Umasuthan Srirangalingam, Helena Gleeson, Melissa Hines, Florian Kurth

**Affiliations:** 1 Department of Women’s and Children’s Health, Uppsala University, Uppsala, Sweden; 2 School of Psychology, University of Auckland, Auckland, New Zealand; 3 Laboratory of Neuro Imaging, USC Stevens Institute for Neuroimaging and Informatics, Keck School of Medicine of USC, University of Southern California, Los Angeles, CA USA; 4 Department of Psychology, University of Deusto, Bilbao, Spain; 5 IKERBASQUE, Basque Foundation for Science, Bilbao, Spain; 6 Department of Psychology, University of Cambridge, Cambridge, UK; 7 Department of Paediatrics, Addenbrooke’s Hospital, University of Cambridge, Cambridge, UK; 8 The Weston Centre for Paediatric Endocrinology and Diabetes, Addenbrooke’s Hospital, University of Cambridge, Cambridge, UK; 9 Department of Endocrinology and Diabetes, University College Hospital London, London, NW1 2BU UK; 10 Queen Elizabeth Hospital, Birmingham, UK; 11 Department of Diagnostic and Interventional Radiology, Jena University Hospital, Jena, Germany

**Keywords:** Androgens, Brain, Cerebral cortex, Corticosteroids, Magnetic resonance imaging, Sex, Diseases, Endocrinology, Medical research, Neuroscience, Physiology

## Abstract

Congenital adrenal hyperplasia (CAH), a genetic condition that disrupts cortisol synthesis, is associated with elevated androgen levels in females with CAH. Altered hormonal milieus have been linked to changes in brain structure, yet little is known about how CAH affects the cerebral cortex. Here, we investigated vertex-wise cortical thickness in 53 individuals with CAH (33 women and 20 men) and 53 sex- and age-matched controls (33 women and 20 men) using surface-based morphometry. There were no significant effects of biological sex and no significant diagnosis-by-sex interaction. However, there was a significant effect of diagnosis, with thinner cortices in various regions across the left and right lateral and medial surfaces in individuals with CAH compared to controls. These findings point to widespread cortical alterations in CAH, independent of sex, and extend prior evidence of structural brain variations in this endocrine disorder. The observed cortical thinning may result from multiple factors, including prenatally reduced cortisol levels, potential long-term consequences of postnatal glucocorticoid treatment, and ongoing physiological and psychosocial stressors.

**Supplementary Information:**

The online version contains supplementary material available at 10.1038/s41598-026-45407-2.

## Introduction

Congenital adrenal hyperplasia (CAH) is a genetic condition with a prevalence of approximately 1 in 16,000 births^1^. CAH predominately results from a mutation of the gene *CYP21A2*, which encodes 21-hydroxylase, an enzyme essential for cortisol synthesis in the adrenal glands. Consequently, both females and males with CAH exhibit reduced prenatal cortisol levels, while females additionally experience androgen levels that exceed typical ranges^2,3^. After birth, glucocorticoid therapy is initiated in males and females to restore cortisol levels. In females, this therapy also normalizes androgen levels.

The prenatal lack of cortisol as well as potential adverse consequences of the lifelong treatment (e.g., glucocorticoid overexposure), may impact brain structure in both sexes^4^. Similarly, the prenatal androgen overexposure in females with CAH could leave an imprint on brain anatomy^5^. Indeed, several neuroimaging studies—including studies based on the current sample^6–8^—point to significant structural brain alterations in individuals with CAH^5,9^. However, research is sparse overall, and findings are inconsistent in terms of the brain region(s) affected. For example, with respect to gray matter—one of the brain’s main tissue types—studies have detected significant reductions in the frontal, temporal, parietal, and occipital cortices; subcortical structures (e.g., hippocampus, amygdala, and thalamus); the cerebellum; and even the brainstem^5,7,9^. Despite evidence of gray matter alterations in CAH overall, research with respect to cortical thickness—a widely used gray matter measure reflecting the width of the cortical ribbon—remains extremely limited. To our knowledge, only one published article exists reporting thinner cortices in individuals with CAH (21 women and 16 men) compared to healthy controls (26 women and 17 men), within the left and right rostral middle frontal gyrus, left superior parietal cortex, and right inferior parietal cortex^10^. In addition, there is a conference abstract reporting thinner cortices in 17 women with CAH compared to 18 healthy control women, within the left and right parahippocampus^11^.

The present study was conducted to extend this understudied field of research, both to explore the potential relevance of cortical thickness as a biomarker of neuroanatomical variation in CAH and to provide insight into how the condition’s atypical hormonal milieu may impact the cerebral cortex. For this purpose, we analyzed high-resolution MRI data from the largest CAH cohort to date (*n* = 53), including both women with CAH (*n* = 33) and men with CAH (*n* = 20). Using a surface-based morphometric approach, we assessed vertex-wise cortical thickness across the entire cortex, without restricting analyses to predefined regions of interest. Based on the aforementioned reports of gray matter reductions and cortical thinning in CAH, we expected thinner cortices in individuals with CAH compared to controls. In addition to assessing the main effect of diagnosis (CAH vs. controls), we also evaluated potential sex effects (women vs. men) as well as diagnosis-by-sex interactions. This was motivated by prior analyses in the same sample demonstrating significant main effects of sex on voxel-wise gray matter^7^ and vertex-wise cortical gyrification^12^, as well as significant diagnosis-by-sex interactions for estimated brain sex^13^.

## Methods

### Participants

Our sample included 53 individuals (33 women and 20 men) with classic CAH^2^, aged between 18 and 45 years (mean ± SD: 30.14.85 ± 7.90 years) and 53 controls (33 women and 20 men), aged between 18 and 45 years (mean ± SD: 30.35 ± 7.67 years). All participants were recruited in the United Kingdom (UK), through National Health Service (NHS) clinics, a national CAH support group, as well as flyers and advertisements posted in hospitals, general practice clinics, or online. Individuals with CAH were matched to controls with respect to sex, age, level of education, and verbal intelligence as measured by the Advanced Vocabulary Test^14^.

All participants were screened to ensure the absence of neurological or psychiatric disorders and any contraindications to magnetic resonance imaging (MRI). Ethical approval for the study was obtained from the National Health Service and the Health Research Authority in the United Kingdom (15/EM/0532), as well as from the University of Auckland in New Zealand (020825). All participants provided their informed consent, and all experiments were performed in accordance with relevant guidelines and regulations.

### Image acquisition and processing

Brain images were acquired on a Siemens 3 Tesla Skyra system with a 32-channel head coil using the following parameters: TR = 2300 ms, TE = 2.98 ms, flip angle = 9°, matrix size = 256 × 240, 176 sagittal sections, voxel size = 1 × 1 × 1 mm^3^. The T1-weighted data were processed in MATLAB 2022b (www.mathworks.com/products/matlab) using SPM12 (version r7771; https://www.fil.ion.ucl.ac.uk/spm/) and CAT12 (version 12.8^15^).

More specifically, all brain images were corrected for magnetic field inhomogeneities and segmented into gray matter (GM), white matter (WM), and cerebrospinal fluid (CSF), as previously described^15^. This was followed by removing the brain stem and cerebellum and by separating the hemispheres. For each hemisphere, the cortical surface was reconstructed, and the vertex-wise cortical thickness was calculated, as detailed elsewhere^16^. The individual surfaces were then spatially normalized to the *FsAverage* template^17^, and the resulting normalized cortical thickness values were smoothed using a 12 mm FWHM kernel.

### Statistical analysis

The statistical analysis was performed using the General Linear Model as implemented in SPM to assess the effects of diagnosis (CAH vs. controls) and biological sex (female vs. male), as well as the diagnosis-by-sex interaction. The dependent variables were the smoothed normalized cortical thickness values, and the independent variables were diagnosis, sex, and the diagnosis-by-sex interaction. Age was treated as a confounding covariate. Significance was established by controlling the family-wise error (FWE) at *p*≤ 0.05 using non-parametric statistics based on threshold-free cluster enhancement (TFCE^18^), as implemented in the TFCE toolbox (r269; https://neuro.uni-jena.github.io/software). Finally, the Desikan Killiany cortical atlas (DK-40^19^) was used to determine the underlying anatomical region(s) pertaining to each significance cluster.

## Results

**Fig. 1:**
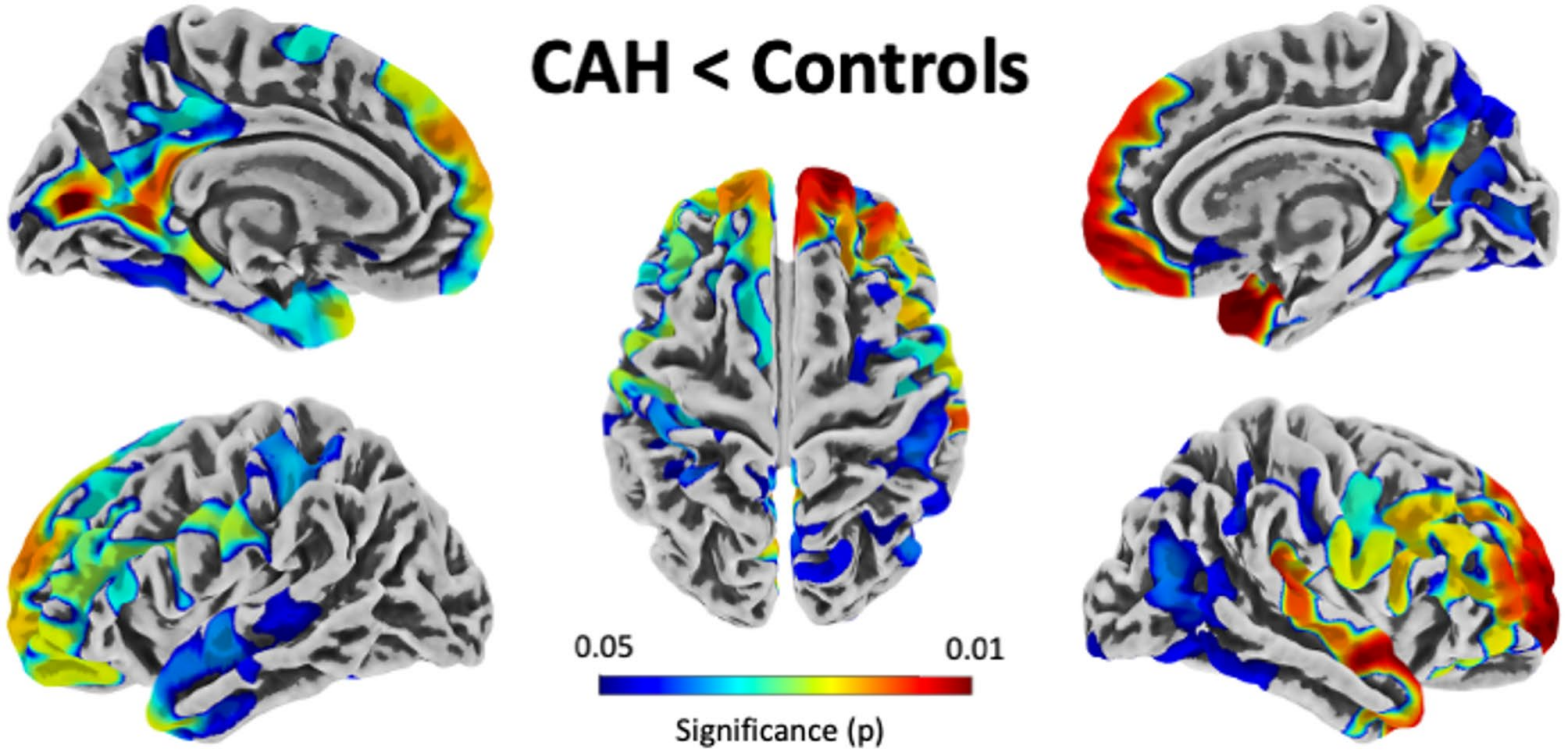
Regions of significantly thinner cortices in individuals with CAH compared to controls (CAH < Controls). The color bar encodes significance (p), FWE-corrected for multiple comparisons. The significance clusters are projected onto the surface of the *FsAverage* template. There was no region where individuals with CAH had significantly thicker cortices than controls.

There was a significant main effect of diagnosis, with thinner cortices in individuals with CAH compared to controls. As shown in Fig. 1, numerous cortical regions were affected, both in the left and right hemisphere, across the lateral and medial surface, and within all four lobes.

In the left hemisphere, there were three significance clusters. The largest cluster (see cluster L-1) contained 23,657 vertices and comprised frontal and parietal regions, specifically the rostral middle frontal gyrus, superior frontal gyrus, postcentral gyrus, precentral gyrus, lateral orbitofrontal gyrus, pars opercularis, pars orbitalis, pars triangularis, supramarginal gyrus, frontal pole, and superior parietal gyrus. For detailed information on each cluster in the left hemisphere, see Table 1 and Supplementary Table 1.

**Table 1:** Left Hemisphere: Significance Clusters (CAH < Controls) as per the DK-40 cortical atlas.

Significance Cluster (in red)		Number of Vertices	Maximum Significance	Atlas Regions (Overlap*)
L-1		23,657	*p* = 0.015	superior frontal (22%), rostral middle frontal (21%), Postcentral (20%), precentral (11%)
L-2		23,215	*p* = 0.008	precuneus (15%), superior temporal (14%), isthmus cingulate (10%)
L-3		96	*p* = 0.049	rostral anterior cingulate (69%), medial orbitofrontal (31%)

*Restricted to areas with ≥10% overlap with the corresponding atlas region (for a complete list of the areas, see Supplementary Table 1).

In the right hemisphere, there were four significance clusters. The largest cluster (see cluster R-1) contained 34,917 vertices and comprised frontal, temporal, and parietal regions, specifically the rostral middle frontal gyrus, superior temporal gyrus, superior frontal gyrus, precentral gyrus, postcentral gyrus, medial orbitofrontal gyrus, supramarginal gyrus, pars triangularis, lateral orbitofrontal gyrus, caudal middle frontal gyrus, pars opercularis, temporal pole, insula, transverse temporal gyrus, pars orbitalis gyrus, inferior temporal gyrus, frontal pole, middle temporal gyrus, and entorhinal gyrus. For detailed information on each cluster in the right hemisphere, see Table 2 and Supplementary Table 2.

**Table 2:** Right Hemisphere: Significance Clusters (CAH < Controls) as per the DK-40 cortical atlas.

Significance Cluster (in red)		Number of Vertices	Maximum Significance	Atlas Regions (Overlap*)
R-1		34,917	*p* = 0.009	rostral middle frontal (19%), superior temporal (12%), supramarginal (11%)
R-2		11,178	*p* = 0.016	precuneus (23%), lingual (18%), isthmus cingulate (16%), pericalcarine (11%), fusiform (11%), superior parietal (10%)
R-3		7,129	*p* = 0.033	inferior parietal (33%), inferior temporal (23%), lateral occipital (21%), middle temporal (13%)
R-4		1,064	*p* = 0.044	inferior parietal (76%), superior parietal (24%)

*Restricted to areas with ≥10% overlap with the corresponding atlas region (for a complete list of the areas, see Supplementary Table 2).

Importantly, no region was significantly thicker in individuals with CAH than in controls. Moreover, there was no significant main effect of sex or diagnosis-by-sex interaction.

## Discussion

To our knowledge, this is only the third study to examine cortical thickness in CAH (and the second full-length published report). We observed a significant main effect of diagnosis, with thinner cortices in individuals with CAH compared to controls. In contrast, there was no significant diagnosis-by-sex interaction or significant main effect of sex.

### Correspondence with findings in independent samples

With respect to the direction of the observed diagnosis effect (CAH < Controls), the present findings are in agreement with the outcomes of the two previously published studies^10,11^, which also reported thinner cortices in individuals with CAH compared to healthy controls. In addition, there is a regional correspondence between our findings and the effects reported by Webb et al.^11^, within the left and right parahippocampus (see current clusters L-2 and R-2) as well as by Van’t Westeinde et al.^10^, within the left and right rostral middle frontal gyrus (see current clusters L-1 and R-1), left superior parietal cortex (see current cluster L-1), and right inferior parietal cortex (see current cluster R-1). Furthermore, when applying a voxel-based approach targeting gray matter in parallel to cortical thickness, Van’t Westeinde et al.^10^, also detected smaller volumes of the left precuneus in CAH (see current cluster L-2). Overall, CAH-related alterations as observed in the current study are more extended than previously observed in frontal, temporal, and parietal regions and additionally evident in occipital regions. Moreover, some effects that were confined to one hemisphere in prior studies (e.g., the precuneus effects on the left side) were evident bilaterally in the present study (e.g., see current cluster R-2 for the precuneus effect on the right side). This could be due to the increased statistical power as our sample included 53 participants with CAH, as opposed to 17 participants^11^ or 37 participants^10^.

### Correspondence with findings in the same sample

In agreement with the current outcomes, we previously reported significant effects of diagnosis (CAH < Controls) when analyzing the same sample with respect to global white matter volume^8^, callosal area and point-wise callosal thickness^6^, as well as voxel-wise gray matter volume^7^. In terms of regional correspondence, the current findings are not directly comparable to the outcomes of the white matter study, as that analysis was global in nature. Comparability is still somewhat limited with respect to the outcomes of the corpus callosum study^6^, although there appears to be some agreement in that significant diagnosis effects were reported specifically within the isthmus and splenium – callosal regions proposed to connect the temporal, parietal, and occipital cortices^20^.

Comparability seems warranted with respect to the outcomes of the voxel-wise gray matter study^7^, which detected significant diagnosis effects within the left and right calcarine cortex (see current clusters L-2 and R-2), the left parahippocampal cortex (see current cluster L-2), and the left lingual gyrus (see current cluster L-2), although we also observed effects in the right lingual gyrus and in several additional regions. The voxel-wise gray matter study^7^ additionally reported bilateral gray matter reductions in CAH within the amygdala, a subcortical structure for which cortical thickness is not defined. Notwithstanding, overall, the current cortical thickness analysis revealed more widespread effects than the gray matter study within the same sample, which might reflect methodological differences between surface-based and voxel-based approaches. It is possible, for example, that cortical thickness measures benefit from more accurate surface-based alignment and reduced partial-volume effects, thereby enhancing sensitivity to subtle and spatially distributed cortical alterations.

### Possible contributors to the observed effects

The observed cortical thinning in individuals with CAH likely reflects a combination of factors. First, there may be direct effects of the condition itself, including the genetic mutation in *CYP21A2* and its broader biochemical consequences^21^, which could influence neural development independently of measured hormone levels. Second, reduced cortisol exposure during prenatal development may alter normal cortical maturation, given the role of glucocorticoids in neuronal proliferation, migration, and synaptic pruning^4^. Third, long-term glucocorticoid treatment after birth, while necessary to replace deficient cortisol, may not perfectly replicate physiological hormone levels and could exert additional effects on the cerebral cortex in particular, or on the brain more generally^22^. In support of this possibility, studies examining the effects of (excess) glucocorticoids outside the framework of CAH, in both humans and animals, have demonstrated resulting brain alterations at both the microscopic and macroscopic levels^23–26^. In addition, individuals with CAH may experience ongoing physiological and psychosocial stressors^27^, arising both from the metabolic demands of the condition and from environmental responses, such as frequent medical interventions, societal pressures, or adaptive challenges in daily life. These stressors could further influence cortical development and maintenance, potentially interacting with genetic and hormonal factors. Importantly, the absence of significant diagnosis-by-sex interactions indicates that the observed cortical thickness alterations in CAH are unlikely to be driven by androgen-specific effects, which would be expected to disproportionately affect females with CAH, given their prenatal androgen overexposure.

### Summary

In summary, the present study provides further evidence that individuals with CAH exhibit widespread cortical thinning compared to healthy controls. These effects are consistent with prior neuroimaging findings in both direction and location, yet appear more extensive, potentially reflecting increased statistical power and/or differences in morphometric methodology. The observed cortical alterations are likely due to a combination of factors, including direct genetic effects, prenatal and postnatal glucocorticoid exposure, and environmental influences such as ongoing physiological and psychosocial stressors, rather than androgen-specific effects.

## Supplementary Information

Below is the link to the electronic supplementary material.

Supplementary Material 1

## Data Availability

The data are not publicly available due to ethical restrictions imposed by the signed consent. Any reasonable request for data access should be made to the corresponding author.
