# Supplementary material for: Reduced cortical thickness in individuals with congenital adrenal hyperplasia (CAH)
Source: Sci Rep. 2026 Mar 25;16:9858. doi: 10.1038/s41598-026-45407-2 (PMC13018268; doi:10.1038/s41598-026-45407-2)
Supplement: Supplementary file 1 — Supplementary Material 1 [file 41598_2026_45407_MOESM1_ESM.docx]

**Supplementary Table 1.** Left Hemisphere: Significance Clusters (CAH < Controls) as per the DK-40 cortical atlas

| Significance Cluster (in red) | | Number of Vertices | Maximum Significance | Atlas Regions (Overlap) |
| --- | --- | --- | --- | --- |
| 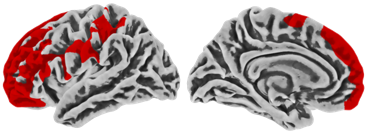L-1 |  | 23,657 | p = 0.015 | superior frontal (22%),  rostral middle frontal (21%),  postcentral (20%),  precentral (11%),  lateral orbitofrontal (6%),  supramarginal (4%),  pars opercularis (4%),  superior parietal (3%),  pars orbitalis (2%),  pars triangularis (2%),  caudal middle frontal (2%),  frontal pole (1%),  medial orbitofrontal (1%) |
| 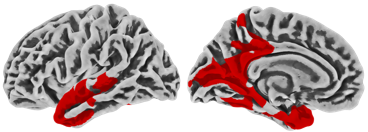L-2 |  | 23,215 | p = 0.008 | precuneus (15%),  superior temporal (14%),  isthmus cingulate (10%),  fusiform (9%),  lingual (8%),  pericalcarine (7%),  parahippocampal (6%),  inferior temporal (5%),  entorhinal (5%),  cuneus (4%),  posterior cingulate (4%),  middle temporal (4%),  temporal pole (4%),  banks sts (2%),  insula (2%),  paracentral (1%) |
| 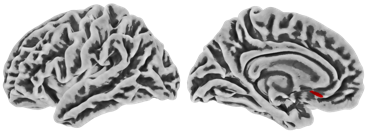L-3 |  | 96 | p = 0.049 | rostral anterior cingulate (69%),  medial orbitofrontal (31%) |

**Supplementary Table 2.** Right Hemisphere: Significance Clusters (CAH < Controls) as per the DK-40 cortical atlas

| Significance Cluster (in red) | | Number of Vertices | Maximum Significance | Atlas Regions (Overlap) |
| --- | --- | --- | --- | --- |
| 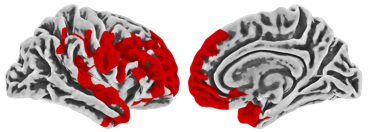R-1 |  | 34,917 | p = 0.009 | rostral middle frontal (19%),  superior temporal (12%),  supramarginal (11%),  precentral (9%),  superior frontal (8%),  postcentral (7%),  caudal middle frontal (5%),  medial orbitofrontal (5%),  pars triangularis (4%),  lateral orbitofrontal (3%),  pars opercularis (3%),  temporal pole (2%),  insula (2%),  superior parietal (2%),  pars orbitalis (1%),  inferior parietal (1%),  transverse temporal (1%),  entorhinal (1%) |
| 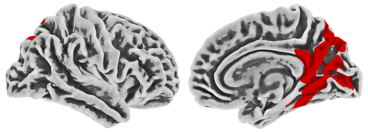R-2 |  | 11,178 | p = 0.016 | precuneus (23%),  lingual (18%),  isthmus cingulate (16%),  pericalcarine (11%),  fusiform (11%),  superior parietal (10%),  cuneus (7%),  parahippocampal (4%) |
| 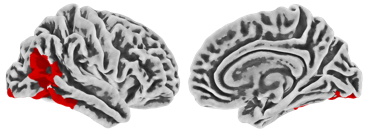R-3 |  | 7,129 | p = 0.033 | inferior parietal (33%),  inferior temporal (23%),  lateral occipital (21%),  middle temporal (13%),  fusiform (5%),  lingual (4%),  banks sts (1%) |
| 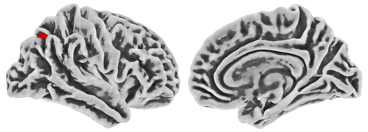R-4 |  | 1,064 | p = 0.044 | inferior parietal (76%),  superior parietal (24%) |
